# The impact of psychiatric utilisation prior to cancer diagnosis on survival of solid organ malignancies

**DOI:** 10.1038/s41416-019-0390-0

**Published:** 2019-03-06

**Authors:** Zachary Klaassen, Christopher J. D. Wallis, Hanan Goldberg, Thenappan Chandrasekar, Rashid K. Sayyid, Stephen B. Williams, Kelvin A. Moses, Martha K. Terris, Robert K. Nam, David Urbach, Peter C. Austin, Paul Kurdyak, Girish S. Kulkarni

**Affiliations:** 1 Department of Surgery, Division of Urology, University of Toronto, University Health Network, Princess Margaret Cancer Centre, Toronto, ON Canada; 2 Institute for Health Policy, Management and Evaluation, University of Toronto, Toronto, ON Canada; 3 Division of Urology, Medical College of Georgia–Augusta University, Augusta, GA USA; 4 Division of Urology, The University of Texas Medical Branch at Galveston, Galveston, TX USA; 5 Department of Urological Surgery, Vanderbilt University Medical Center, Nashville, TN USA; 6 Division of Urology, Sunnybrook Health Sciences Centre, Toronto, ON Canada; 7 Institute for Clinical Evaluative Sciences, Toronto, ON Canada; 8 Department of Surgery, University of Toronto, Women’s College Hospital, Toronto, ON Canada; 9 Institute for Mental Health Policy Research, Centre for Addiction and Mental Health, Toronto, ON Canada

**Keywords:** Cancer epidemiology, Cancer therapy

## Abstract

**Background:**

Among patients with cancer, prior research suggests that patients with mental illness may have reduced survival. The objective was to assess the impact of psychiatric utilisation (PU) prior to cancer diagnosis on survival outcomes.

**Methods:**

All residents of Ontario diagnosed with one of the top 10 malignancies (1997–2014) were included. The primary exposure was psychiatric utilisation gradient (PUG) score in 5 years prior to cancer: 0: none, 1: outpatient, 2: emergency department, 3: hospital admission. A multivariable, cause-specific hazard model was used to assess the effect of PUG score on cancer-specific mortality (CSM), and a Cox proportional hazard model for effect on all-cause mortality (ACM).

**Results:**

A toal of 676,125 patients were included: 359,465 (53.2%) with PUG 0, 304,559 (45.0%) PUG 1, 7901 (1.2%) PUG 2, and 4200 (0.6%) PUG 3. Increasing PUG score was independently associated with worse CSM, with an effect gradient across the intensity of pre-diagnosis PU (vs PUG 0): PUG 1 h 1.05 (95% CI 1.04–1.06), PUG 2 h 1.36 (95% CI 1.30–1.42), and PUG 3 h 1.73 (95% CI 1.63–1.84). Increasing PUG score was also associated with worse ACM.

**Conclusions:**

Pre-cancer diagnosis PU is independently associated with worse CSM and ACM following diagnosis among patients with solid organ malignancies.

## Background

Cancer is the second leading cause of mortality in the United States and Canada and its association with morbidity, including psychiatric disease, is well established.^1^ However, less studied is the effect of psychiatric disease on cancer outcomes. Previous work has suggested that psychiatric patients may present with higher stage disease, are less likely to be treated with appropriate surgery/radiotherapy/chemotherapy, and have poorer cancer-specific survival compared to the general population.^2–10^ As more than 15% of Americans report significant mental illness (major depressive episodes, bipolar disorder, generalised anxiety disorder, or substance abuse),^11^ further characterisation of survival outcomes for these patients when subsequently diagnosed with cancer are needed.

Psychiatric service utilisation can serve as a surrogate for psychiatric comorbidity, including comorbidity severity.^12,13^ As such, the objective of this study was to assess the effect of pre-cancer diagnosis psychiatric utilisation (i.e., the severity of psychiatric comorbidity) on cancer-specific mortality (CSM) and all-cause mortality (ACM). Our primary hypothesis is that patients utilising psychiatric resources prior to cancer diagnosis will encompass an ‘at risk’ population and have poorer survival compared to those without psychiatric utilisation.

## Materials and methods

We conducted a population-based, retrospective cohort study of patients diagnosed with one of the 10 most prevalent malignancies (prostate, breast, colorectal, melanoma, lung, bladder, endometrial, thyroid, kidney, oral; Supplement) in Ontario, Canada (population ~14 million), between January 1997 and December 2014. In Ontario, essential medical care is reimbursed by a single, government-operated health insurance system (Ontario Health Insurance Plan (OHIP)) enabling capture of the entire adult population.

This study was designed and conducted according to Strengthening the Reporting of Observational Studies in Epidemiology guidelines,^14^ and Reporting of Studies Conducted Using Observational Routinely-Collected Health Data Statement.^15^ The University of Toronto Research Ethics Board approved this study.

### Data sources

These datasets were linked using unique encoded identifiers and analysed at the Institute for Clinical Evaluative Sciences (ICES). The validated databases used are listed in the Supplement.

### Study patients

We identified all residents of Ontario ≥18 years of age with one of the aforementioned malignancies during the study interval (1997–2014) using the Ontario Cancer Registry (OCR). The index date was defined as each patient’s cancer diagnosis date. Patients were considered eligible if their first cancer diagnosis was one of the 10 evaluated.

Using linked administrative databases, we collected demographic information, including age at cancer diagnosis (continuous), gender, socioeconomic status (operationalised as quintile of median neighbourhood income), rurality (yes vs no), year of diagnosis (by tertiles), and general comorbidity (Johns Hopkins aggregate disease group^16^), operationalised as low (≤5), intermediate (6–9), and high (≥10). The Johns Hopkins aggregate disease group is based on previous healthcare utilisation and has better discrimination than the Charlson score in comorbidity assessment.^17^

### Exposure

The primary exposure was psychiatric utilisation (PU) during the 5 years prior to cancer diagnosis, operationalised categorically. Specifically, the psychiatric utilisation gradient (PUG) score was defined as: 0: no psychiatric utilisation; 1: outpatient psychiatric utilisation (captured together as a visit to a psychiatrist or primary care provider for a psychiatric condition); 2: emergency department visit for psychiatric utilisation; 3: hospital admission for psychiatric utilisation; PUG score 2 and 3 classified as 'acute care utilisation'. Patients received a PUG score based on their highest level of psychiatric utilisation resulting in mutually exclusive exposure categories: i.e., a person receiving outpatient psychiatric utilisation and admitted to the hospital for psychiatric purposes was given a PUG score of 3. Levels of psychiatric utilisation used to generate the PUG score were identified using a combination of OHIP outpatient and hospital billing codes as previously described.^12,13^

### Outcomes

The primary outcome was CSM, defined as death associated with the patient’s primary cancer diagnosis. Oncologic causes of death have been validated in the OCR.^18,19^ The secondary outcome was ACM, determined from the Registered Persons database. The final day of follow-up was 29 September 2017.

### Statistical analysis

Patients’ demographic and clinical variables were compared, stratified by PUG score. Continuous variables were summarised using median and interquartile ranges (IQR) and compared between groups using the Kruskal–Wallis test. Categorical variables were reported as proportions and compared using the Chi-square test. Cumulative incidence functions were used to assess the cumulative incidence of CSM over time, while Kaplan–Meier survival curves were used to estimate the incidence of ACM over time. These analyses were stratified by PUG score. To assess the effect of PUG score on CSM, we used a multivariable cause-specific hazard model, using a priori variable selection adjusting for age at diagnosis, gender, Aggregated Diagnosis Groups (ADG) comorbidity score (The Johns Hopkins ACG (R) System version 10.0), income quintile, rurality, and year of diagnosis. To assess the effect of PUG score on ACM, we used Cox proportional hazards model, adjusting for the same variables. The assumptions underlying the models were assessed and no violations were identified.

### Sensitivity analysis

We conducted several pre-planned sensitivity analyses. First, we repeated the analysis among patients diagnosed from 2007 onwards for whom disease stage data were available (*n* = 111,620), also adjusting for the effect of cancer stage. Second, to assess the impact of timing of psychiatric utilisation prior to cancer diagnosis, psychiatric utilisation was operationalised ≤12 months vs >12 months (all within 5 years) prior to cancer diagnosis. Third, given that the PUG score is not a validated metric, we performed a sensitivity analysis using pre-cancer diagnosis of schizophrenia/schizoaffective disorder (*n* = 3199), since the coding for identifying these patients has previously been validated using ICES databases.^12^ Patients with schizophrenia/schizoaffective disorder were compared to PUG score 0 patients without schizophrenia/schizoaffective disorder. We chose patients with PUG 0 without schizophrenia/schizoaffective disorder as the comparator considering that these patients have no psychiatric utilisation and are the comparator for the other analyses. Finally, the effect of PUG score on mortality was assessed within each of the 10 primary malignancies. All sensitivity analyses were cause-specific hazard models (cancer-specific outcomes) or Cox proportional hazards models (overall outcomes), adjusted for the same variables as the full models.

Statistical significance was set at *p* < 0.05 based on two-tailed comparison. All analyses were performed using SAS version 9.4 (SAS Institute Inc.).

## Results

A total of 676,125 patients were included in the analysis: 359,465 (53.2%) with PUG score 0, 304,559 (45.0%) with PUG score 1, 7901 (1.2%) with PUG score 2, and 4200 (0.6%) with PUG score 3. Patients with increasing PUG score were generally younger, more often female, had higher ADG comorbidity score, had lower income, lived in non-rural communities, and received a cancer diagnosis in more recent years (all *p* < 0.0001; Table 1).

**Table 1:** Baseline characteristics of patients with cancer stratified by pre-cancer diagnosis psychiatric utilisation gradient

	No. (%)				
Characteristic	PUG Score 0	PUG Score 1	PUG Score 2	PUG Score 3	*P* value
Sample size	359,465	304,559	7901	4200	
Age at diagnosis, y					<0.0001
Median (IQR)	67 (58–75)	66 (56–75)	62 (52–74)	61 (53–71)	
Gender					<0.0001
Male	196,828 (54.8)	138,736 (45.5)	3111 (39.4)	1734 (41.3)	
Female	162,637 (45.2)	165,823 (54.5)	4790 (60.6)	2466 (58.7)	
Income quintile					<0.0001
1: Lowest	64,388 (17.9)	58,233 (19.1)	2151 (27.2)	1343 (32.0)	
2	72,577 (20.2)	61,995 (20.4)	1747 (22.1)	869 (20.7)	
3	71,439 (19.9)	59,994 (19.7)	1499 (19.0)	729 (17.4)	
4	73,662 (20.5)	60,951 (20.0)	1289 (16.3)	679 (16.2)	
5: Highest	77,399 (21.5)	63,386 (20.8)	1215 (15.4)	580 (13.7)	
Comorbidity (ADG category)					<0.0001
Low	151,830 (42.2)	58,479 (19.2)	1062 (13.4)	503 (12.0)	
Intermediate	147,635 (41.1)	133,093 (43.7)	2875 (36.4)	1361 (32.4)	
High	60,000 (16.7)	112,987 (37.1)	3964 (50.2)	2336 (55.6)	
Rurality					<0.0001
Yes	59,069 (16.4)	39,450 (13.0)	1447 (18.3)	561 (13.4)	
Cancer anatomic site					<0.0001
Prostate (*n* = 137,699)	80,625 (22.4)	55,646 (18.3)	950 (12.0)	478 (11.4)	
Breast (*n* = 131,610)	64,130 (17.8)	64,883 (21.3)	1656 (21.0)	941 (22.4)	
Lung (*n* = 122,822)	60,675 (16.9)	59,170 (19.4)	1935 (24.5)	1102 (26.2)	
Colorectal (*n* = 119,180)	67,211 (18.7)	50,168 (16.5)	1160 (14.7)	641 (15.3)	
Melanoma (*n* = 41,708)	23,293 (6.5)	17,716 (5.8)	462 (5.9)	237 (5.6)	
Thyroid (*n* = 30,554)	14,213 (4.0)	15,597 (5.1)	538 (6.8)	206 (4.9)	
Bladder (*n* = 29,884)	16,579 (4.6)	12,860 (4.2)	299 (3.8)	146 (3.5)	
Endometrial (*n* = 28,346)	15,108 (4.2)	12,721 (4.2)	350 (4.4)	167 (4.0)	
Kidney (*n* = 23,485)	11,950 (3.3)	11,047 (3.6)	347 (4.4)	141 (3.4)	
Oral (*n* = 10,777)	5681 (1.6)	4751 (1.6)	204 (2.6)	141 (3.4)	
Year of cancer diagnosis					<0.0001
1997–2002	102,499 (28.5)	94,127 (30.9)	415 (5.3)	257 (6.1)	
2003–2008	118,806 (33.1)	103,014 (33.8)	3290 (41.6)	1747 (41.6)	
2009–2014	138,160 (38.4)	107,418 (35.3)	4196 (53.1)	2196 (52.3)	
AJCC Stage^a^					<0.0001
I	20,317 (33.0)	17,350 (36.7)	605 (35.0)	308 (32.9)	
II	12,875 (20.9)	8928 (18.9)	235 (13.6)	107 (11.4)	
III	6041 (9.8)	3985 (8.4)	134 (7.8)	66 (7.1)	
IV	22,434 (36.4)	17,024 (36.0)	756 (43.7)	455 (48.6)	

*PUG* psychiatric utilisation gradient, *IQR* interquartile range, *ADG* Aggregated Diagnosis Groups, *AJCC* American Joint Committee on Cancer

^a^*N* = 111,620 available data

### Primary and secondary outcome analyses

**Fig. 1:**
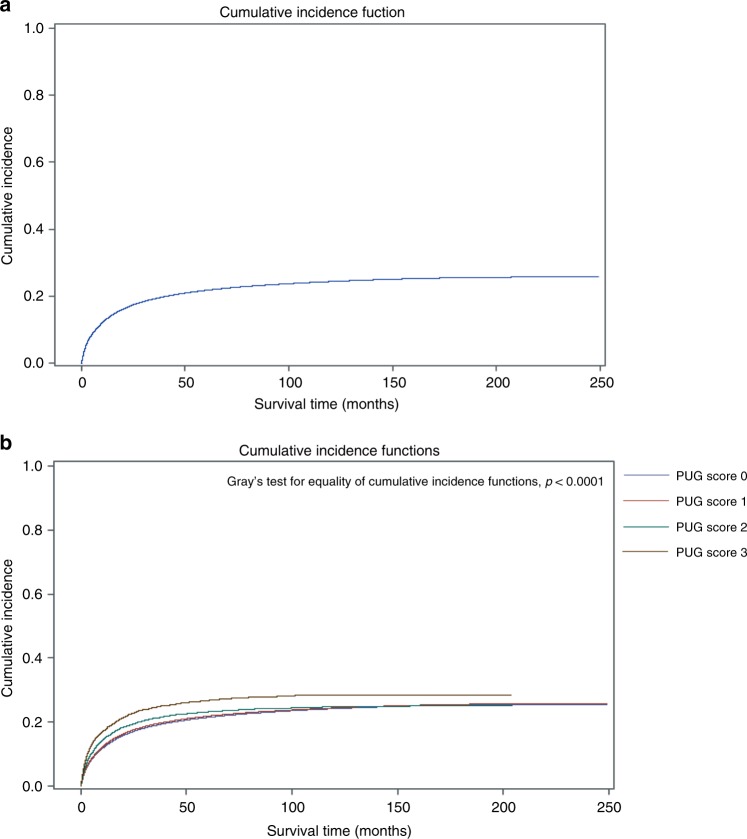
Cumulative incidence function for cancer-specific mortality among patients with cancer (**a**) stratified by psychiatric utilisation gradient (PUG) score (**b**)

The overall 1-, 2-, 5-, and 10-year CSM probabilities were 13.1, 17.2, 21.7, and 24.4%, respectively. CSM differed significantly according to PUG score at the time of cancer diagnosis (Table 2; Fig. 1; Gray’s test for equality of cumulative incidence functions *p* < 0.0001). Among those dying of cancer, the median time from diagnosis to CSM for PUG score 0 patients was 10.3 months (IQR 2.8–28.1), 9.8 months (IQR 2.8–27.2) for those with PUG score 1, 7.0 months (IQR 2.1–18.6) for those with PUG score 2, and 5.9 months (IQR 1.7–18.1) for those with PUG score 3. Increasing PUG score was independently associated with worse CSM after adjusting for age at diagnosis, gender, ADG comorbidity score, income quintile, rurality, and year of diagnosis, with an effect gradient across the intensity of pre-diagnosis psychiatric utilisation (Table 2).

**Table 2:** Multivariable cause-specific hazard analysis (CSM) and Cox proportional hazard analysis (ACM) among patients with cancer

	Cancer-specific mortality		All-cause mortality	
Variable	HR	95% CI	HR	95% CI
PUG score				
PUG 0	Ref	Ref	Ref	Ref
PUG 1	1.05	1.04–1.06	1.04	1.03–1.05
PUG 2	1.36	1.30–1.42	1.44	1.39–1.49
PUG 3	1.73	1.63–1.84	1.94	1.86–2.02
Age	1.034	1.034–1.035	1.053	1.053–1.054
Gender				
Female	Ref	Ref	Ref	Ref
Male	1.07	1.06–1.08	1.12	1.11–1.13
ADG comorbidity score				
Low	Ref	Ref	Ref	Ref
Intermediate	0.90	0.89–0.91	0.96	0.95–0.97
High	0.96	0.95–0.97	1.12	1.11–1.13
Income quintile				
1: Lowest	Ref	Ref	Ref	Ref
2	0.87	0.86–0.89	0.89	0.88–0.89
3	0.82	0.81–0.83	0.84	0.83–0.84
4	0.75	0.74–0.77	0.77	0.77–0.78
5: Highest	0.66	0.65–0.67	0.69	0.68–0.70
Rurality				
No	Ref	Ref	Ref	Ref
Yes	1.06	1.04–1.07	1.09	1.08–1.10
Year of diagnosis				
1997–2002	Ref	Ref	Ref	Ref
2003–2008	0.83	0.82–0.84	0.87	0.87–0.88
2009–2014	0.53	0.53–0.54	0.80	0.80–0.81

*CSM* cancer-specific mortality, *ASM* all-cause mortality, *HR* hazard ratio, *CI* confidence interval, *PUG* psychiatric utilisation gradient, *ADG* Aggregated Diagnosis Groups

**Fig. 2:**
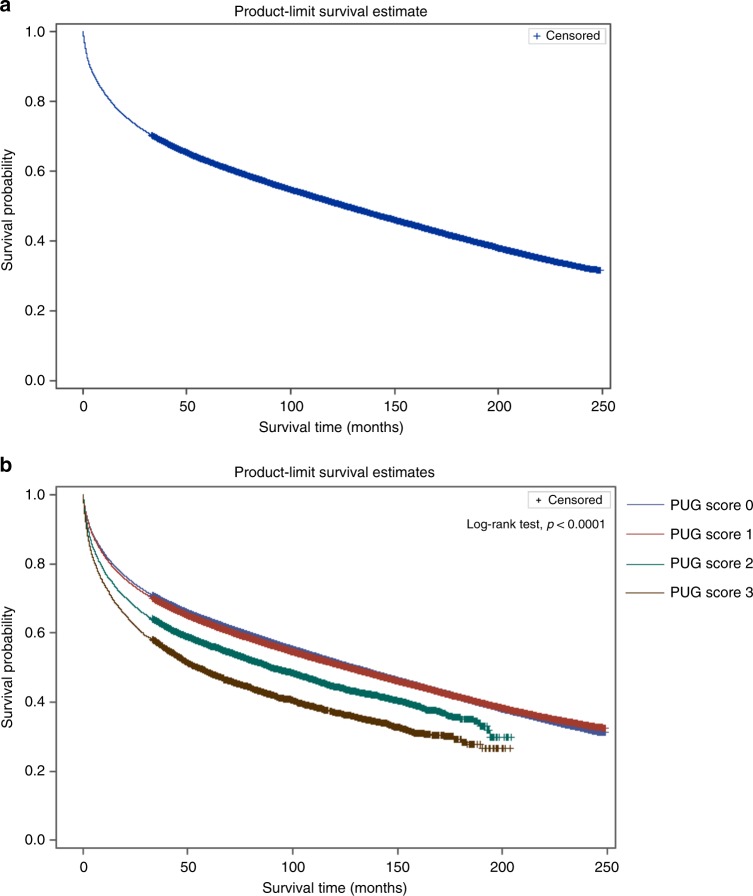
Product-limit survival estimate for overall survival among patients with cancer (**a**) stratified by psychiatric utilisation gradient (PUG) score (**b**)

The overall 1-, 2-, 5-, and 10-year ACM probabilities were 18.9, 26.1, 37.1, and 48.8%, respectively. ACM differed significantly according to PUG score at the time of cancer diagnosis (Table 2; Fig. 2; log-rank test *p* < 0.0001). Among those who died, the median time from cancer diagnosis to death for PUG score 0 patients was 22.2 months (IQR 5.4–65.1), 21.6 months (IQR 5.3–64.4) for those with PUG score 1, 14.2 months (IQR 3.5–40.5) for those with PUG score 2, and 13.6 months (IQR 3.2–39.0) for those with PUG score 3. Increasing PUG score was also independently associated with worse ACM with a similar effect gradient as CSM for intensity of psychiatric utilisation (Table 2).

### Sensitivity analyses for CSM

In a sensitivity analysis adjusting for *cancer stage* (*n* = 111,620), the association between increasing PUG score and worse CSM persisted: compared to PUG score 0, patients with PUG score 1 had an increased risk of CSM (hazard ratio (HR) 1.09, 95% confidence interval (CI) 1.06–1.11), as did those with PUG score 2 (HR 1.35, 95% CI 1.24–1.47), and PUG score 3 (HR 1.40, 95% CI 1.26–1.56). The effect of PUG score sub-stratified by stage is provided in Supplementary Table 1.

When assessing the effect of timing of psychiatric utilisation, patients with PUG scores 1 and 2 had significantly worse CSM if psychiatric services were utilised ≤12 months compared to >12 months prior to cancer diagnosis (Supplementary Table 2). The effect of timing of psychiatric utilisation was not present for patients with PUG score 3.

Cancer patients with schizophrenia/schizoaffective disorder had increased CSM compared to those with PUG score 0: PUG score 1: HR 1.76, 95% CI 1.64–1.88; PUG score 2: HR 2.08, 95% CI 1.86–2.33; PUG score 3: HR 2.02, 95% CI 1.86–2.21.

Bladder and colorectal cancer patients had significantly worse CSM with an effect gradient across the intensity of pre-diagnosis psychiatric utilisation (Supplementary Table 3). Prostate, breast, and lung cancer patients had worse CSM for PUG scores 2 and 3, but not for PUG score 1. Patients with thyroid cancer had worse CSM for PUG score 3, while patients with oral cancer had worse CSM for PUG score 2. For virtually all cancer subtypes, there appeared to be an effect gradient for PUG score on survival outcome for CSM. Given the worse CSM with increasing PUG score gradient for bladder and colorectal cancer, we performed an exploratory analysis to ensure these two cancer types were not responsible for the overall results by limiting the cohort to patients diagnosed with the eight remaining malignancies (*n* = 527,061). Adjusting the models for the same covariates, the overall findings persisted (vs PUG score 0): PUG score 1 (HR 1.04, 95% CI 1.03–1.06), PUG score 2 (HR 1.39, 95% CI 1.33–1.47), and PUG score 3 (HR 1.70, 95% CI 1.59–1.82).

### Sensitivity analyses for ACM

Results of the sensitivity analyses for ACM showed comparable trends in outcomes as the sensitivity analyses for CSM, specifically for cancer stage, timing of psychiatric utilisation, and patients with schizophrenia/schizoaffective disorder (data not shown). When models assessing the effect of PUG score on ACM were performed separately within each malignancy, there was a general trend towards worse risk of survival across increasing PUG scores for all malignancies (Supplementary Table 4).

## Discussion

In this population-based cohort study among 676,125 patients diagnosed with common malignancies in Ontario, Canada, utilisation of psychiatric healthcare services prior to cancer diagnosis was associated with worse CSM and ACM compared to patients with no psychiatric utilisation, with an effect gradient across the intensity of psychiatric utilisation. In fact, acute care utilisation was the strongest predictors of CSM (PUG score 2: HR 1.36; PUG score 3: HR 1.73) and ACM (PUG score 2: HR 1.44; PUG score 3: HR 1.94). This effect persisted when tested among patients with available cancer stage data, a surrogate for burden of disease at diagnosis. The effect of psychiatric utilisation was greater when the time between psychiatric care and cancer diagnosis was shorter. Finally, this effect was particularly pronounced among patients with bladder and colorectal cancer.

Previous studies assessing mental illness and cancer-specific outcomes suggest that these patients likely have a comparable incidence of cancer compared to the general population but may be more likely to present with advanced disease and die of malignancy.^2,3,5,6,8–10,20–23^ However, many of these studies primarily focus on patients with schizophrenia,^10,20–23^ and may not be generalisable to patients with other psychiatric comorbidities. This study is the first to our knowledge to assess the effect of an intensity gradient for psychiatric utilisation (as a surrogate for psychiatric diagnosis severity) and subsequent survival outcomes, regardless of psychiatric diagnosis.

Notably, nearly half of all oncology patients were assessed in a psychiatric outpatient setting, as well as 2 out of every 100 cancer patients either treated in an emergency department setting or hospitalised for a psychiatric condition prior to a cancer diagnosis. This subgroup of cancer patients is at substantially increased risk of CSM from potentially modifiable non-oncologic risk factors. Further, patients receiving outpatient or emergency department psychiatric care ≤12 months prior to diagnosis had a significantly higher risk of CSM compared to those patients receiving similar care 1–5 years prior to diagnosis. Interestingly, timing of hospital admission for psychiatric causes (PUG score 3) prior to diagnosis was not associated with worse survival. Taken together, these results suggest that specific attention should be taken to the acuity of psychiatric utilisation prior to cancer diagnosis. There appears to be an effect on CSM among patients with recent (≤12 months prior to diagnosis) outpatient and emergency department psychiatric utilisation suggesting a crucial 'acute phase' window for these patients above and beyond utilisation at any point during the 5-year period prior to diagnosis. On the contrary, and perhaps unsurprisingly, due to the severity of their mental illness, the timing of psychiatric hospitalisation in the preceding 5-year period prior to cancer diagnosis was not predictive of survival, pointing to the overall poor prognosis in this group of patients regardless of timing of admission.

When assessing the effect of pre-diagnosis psychiatric utilisation on CSM for each malignancy individually, several cancers demonstrated worse CSM across the PUG gradient. In particular, bladder and colorectal cancer patients with psychiatric admissions were particularly at risk of cancer death compared to patients without psychiatric utilisation (HRs, colorectal: 1.71; bladder: 2.18). Previous studies have also demonstrated worse outcomes for psychiatric patients with bladder and colorectal cancer.^2,9,24,25^ Additionally, patients with prostate, breast, and lung cancer had worse CSM for acute care utilisation, but not for PUG score 1. The mechanism for these findings is unclear; however, speculatively, particularly for breast and prostate cancer, these diseases are intimately associated with sexual well-being. Diagnosis and treatment often affects psychosocial quality of life^26^ and thus may be particularly challenging for those with a history of acute psychiatric care utilisation resulting in poor CSM. Kisely et al.^2^ found that psychiatric patients had a 38% increased risk of CSM for non-prostate urologic malignancies and a 54% increased risk for colorectal cancer, compared to the general population. Using SEER-Medicare data, Baillargeon et al.^25^ reported that colorectal patients with pre-existing mental disorders had a 23% increased risk of death compared to patients without mental disorders. Additionally, patients with lung cancer in our study had worse CSM for psychiatric emergency and hospital admission utilisation. Previous studies in patients with schizophrenia have demonstrated a similar association.^10,20^ Lung cancer patients in the Veterans Affairs system with schizophrenia had a 33% increased mortality risk compared to patients without schizophrenia,^10^ which is corroborated in other population-level studies.^20^

There are several hypotheses to explain the association between pre-cancer diagnosis psychiatric utilisation and worse CSM. First, the stress induced from a major psychiatric diagnosis may result in biologic changes that portend a worse cancer diagnosis. Among patients with bladder cancer, Lin et al.^27^ demonstrated that while patients with depressive symptoms were at increased risk of CSM (HR 1.83), this was magnified if they also had short telomere length (HR 3.96). Additionally, major depressive disorder is associated with abnormalities in stress-related biologic systems, which may affect immune surveillance of tumours.^28^ Second, patients with psychiatric comorbidities may be less likely to adhere to follow-up schedules and more likely to engage in behaviours, such as alcoholism and smoking, that are detrimental to overall health and ability to combat cancer.^29^ Third, although speculative, patients with psychiatric comorbidities may not be receiving adequate screening (i.e., colonoscopy for colorectal cancer) and timely or appropriate investigation of cardinal presentations (i.e., haematuria for bladder cancer). Finally, patients with psychiatric comorbidities may be marginalised and receive substandard care that deviates from established guidelines. For instance, patients with psychiatric comorbidities and breast cancer are more likely to experience deviations in stage-specific treatments, resulting in poor 5-year disease recurrence rates.^7^

Regardless of the aetiology for worse CSM and specific psychiatric diagnoses, our study suggests that patients with a psychiatric-specific admission within 5 years of cancer diagnosis, and patients with outpatient or emergency department psychiatric utilisation, specifically within 12 months of cancer diagnosis, require additional vigilance from the healthcare team to ensure comparable stage-specific outcomes can be achieved for all patients. A thorough psychiatric history with documentation of recent psychiatric utilisation and medications may contribute to increased quality of care for newly diagnosed cancer patients. Particularly for these high-risk patients, we feel it is important for the entire healthcare team to ensure they are receiving timely treatment after diagnosis, maintaining guideline specific follow-up regimens, and are located if/when appointments are missed.

To our knowledge, this is the first study assessing the effect of pre-cancer diagnosis psychiatric utilisation on CSM and ACM among patients with prevalent solid organ malignancies. In addition to the large sample size, this study has significant strengths owing to its population-based nature. First, this study was performed in Ontario, Canada, a jurisdiction in which all relevant health services are available free of direct cost and are systematically tracked in administrative databases. Second, as all patients with the included malignancies in Canada’s largest province were identified over a nearly two-decade timeframe, these results are generalisable, representing the population spectrum of oncologic clinical practice. Third, all outpatient, emergency department visits and hospital admissions occurring anywhere in the province of Ontario were captured, reducing ascertainment bias.

This study has several limitations. First, we were unable to account for the effect of specific treatment modalities. However, given the robustness of the findings when limiting to patients with stage data, a significant effect of treatment is unlikely. Variations in treatment may also be on the causal pathway of the association described herein, with deviations in treatment in higher PUG score patients leading to worse outcomes. Correcting for treatment may thus inappropriately bias the findings to the null. Second, we limited the inclusion criteria to the 10 most prevalent malignancies in Canada, thus these results may not be generalisable to patients with haematologic malignancies or rare solid organ malignancies. Third, while the PUG score has face validity to represent psychiatric severity and utilisation,^12,13^ it requires further validation. Fourth, we did not account for whether PUG 1 patients were also more likely to seek treatment from their primary care provider for general medical conditions. However, given that PUG 1 patients incurred worse CSM compared to PUG 0 patients, we feel that this is unlikely to bias outcomes. Finally, although we performed several sensitivity analyses to enhance the robustness of the results, residual confounding is a possibility. Epidemiological studies are designed to identify associations, and further work is necessary to understand causal pathways.

## Conclusion

Among adults with prevalent solid organ malignancies in Ontario, Canada, pre-cancer diagnosis psychiatric utilisation is independently associated with worse CSM and ACM. Patients with intense psychiatric utilisation prior to diagnosis are more likely to suffer CSM compared to patients without psychiatric utilisation. The specific factors underlying the observed associations remain to be elucidated.

## Supplementary information

Supplementary Table 1

Supplementary Table 2

Supplementary Table 3

Supplementary Table 4

Supplement

## Data Availability

The data for this manuscript were obtained from the Institute of Clinical Evaluative Sciences in Toronto, Ontario, Canada *CENTRAL* location at the Sunnybrook Health Sciences Center. The project TRIM number was 2018 0990 009 000 and permission to use the data was granted by the Cancer ICES Research Program and the University of Toronto Research Ethics Board. Z.K. had full access to the data. The data that support the findings of this study are available from the Cancer ICES Research Program, but restrictions apply to the availability of these data, which were used under license for the current study, and so are not publicly available. Data are however available from the authors upon reasonable request and with permission of the Cancer ICES Research Program.
