# Supplementary material for: The impact of psychiatric utilisation prior to cancer diagnosis on survival of solid organ malignancies
Source: Br J Cancer. 2019 Mar 6;120(8):840–7. doi: 10.1038/s41416-019-0390-0 (PMC6474265; doi:10.1038/s41416-019-0390-0)
Supplement: Supplementary file 1 — Supplementary Table 1 [file 41416_2019_390_MOESM1_ESM.docx]

**Supplementary Table 1:** Multivariable cause-specific hazards analysis for CSM stratified by cancer stage

| **Stage** | **n** | **HR** | **95%CI** | **p-value** |
| --- | --- | --- | --- | --- |
| *1* | 38,580 |  |  | <0.0001 |
| PUG Score 0 |  | Ref | Ref |  |
| PUG Score 1 |  | 1.15 | 0.99-1.33 |  |
| PUG Score 2 |  | 2.25 | 1.50-3.39 |  |
| PUG Score 3 |  | 1.72 | 0.81-3.64 |  |
| *2* | 22,145 |  |  | <0.0001 |
| PUG Score 0 |  | Ref | Ref |  |
| PUG Score 1 |  | 0.91 | 0.79-1.05 |  |
| PUG Score 2 |  | 0.94 | 0.47-1.90 |  |
| PUG Score 3 |  | 2.31 | 1.09-4.88 |  |
| *3* | 10,226 |  |  | <0.0001 |
| PUG Score 0 |  | Ref | Ref |  |
| PUG Score 1 |  | 1.02 | 0.87-1.19 |  |
| PUG Score 2 |  | 1.35 | 0.79-2.32 |  |
| PUG Score 3 |  | 2.52 | 1.29-4.91 |  |
| *4* | 40,669 |  |  | <0.0001 |
| PUG Score 0 |  | Ref | Ref |  |
| PUG Score 1 |  | 1.09 | 1.07-1.12 |  |
| PUG Score 2 |  | 1.31 | 1.20-1.44 |  |
| PUG Score 3 |  | 1.36 | 1.21-1.52 |  |
| Models adjusted for age at diagnosis, gender, ADG comorbidity, income quintile, rurality, year of diagnosis | | | |  |
