# Supplementary material for: The impact of psychiatric utilisation prior to cancer diagnosis on survival of solid organ malignancies
Source: Br J Cancer. 2019 Mar 6;120(8):840–7. doi: 10.1038/s41416-019-0390-0 (PMC6474265; doi:10.1038/s41416-019-0390-0)
Supplement: Supplementary file 2 — Supplementary Table 2 [file 41416_2019_390_MOESM2_ESM.docx]

**Supplementary Table 2:** Multivariable cause-specific hazards analysis for CSM stratified by timing of pre-diagnosis psychiatric utilization

| **PUG Score** | **n** | **HR** | **95%CI** |
| --- | --- | --- | --- |
| *PUG 1* |  |  |  |
| >12 months | 260,457 | Ref | Ref |
| ≤12 months | 44,102 | 1.11 | 1.09-1.14 |
| *PUG 2* |  |  |  |
| >12 months | 5,739 | Ref | Ref |
| ≤12 months | 2,162 | 1.19 | 1.08-1.32 |
| *PUG 3* |  |  |  |
| >12 months | 3,356 | Ref | Ref |
| ≤12 months | 844 | 1.06 | 0.92-1.22 |
| Models adjusted for age at diagnosis, gender, ADG comorbidity, income quintile, rurality, year of diagnosis | | | |
