# Supplementary material for: The impact of psychiatric utilisation prior to cancer diagnosis on survival of solid organ malignancies
Source: Br J Cancer. 2019 Mar 6;120(8):840–7. doi: 10.1038/s41416-019-0390-0 (PMC6474265; doi:10.1038/s41416-019-0390-0)
Supplement: Supplementary file 3 — Supplementary Table 3 [file 41416_2019_390_MOESM3_ESM.docx]

**Supplementary Table 3:** Anatomic site-specific multivariable cause-specific hazards analysis for CSM assessing the effect of psychiatric utilization

|  | **HR (95%CI)** | | | |
| --- | --- | --- | --- | --- |
| **Anatomic Site** | **PUG Score 0** | **PUG Score 1** | **PUG Score 2** | **PUG Score 3** |
| Prostate | Ref | 0.98 (0.94-1.01) | 1.28 (1.01-1.62) | 2.23 (1.69-2.94) |
| Breast | Ref | 0.99 (0.96-1.02) | 1.20 (1.03-1.40) | 1.80 (1.53-2.13) |
| Lung* | Ref | 1.00 (0.99-1.02) | 1.17 (1.11-1.24) | 1.26 (1.17-1.37) |
| Colorectal* | Ref | 1.04 (1.02-1.07) | 1.15 (1.01-1.31) | 1.71 (1.47-1.99) |
| Melanoma* | Ref | 0.95 (0.89-1.02) | 0.97 (0.69-1.37) | 1.37 (0.91-2.07) |
| Thyroid* | Ref | 0.95 (0.81-1.12) | 1.70 (0.84-3.46) | 2.71 (1.11-6.61) |
| Bladder* | Ref | 1.09 (1.03-1.14) | 1.29 (1.02-1.64) | 2.18 (1.62-2.93) |
| Endometrial | Ref | 1.07 (0.97-1.18) | 1.00 (0.61-1.64) | 1.37 (0.68-2.76) |
| Kidney* | Ref | 0.98 (0.93-1.04) | 0.75 (0.55-1.02) | 0.80 (0.48-1.33) |
| Oral* | Ref | 1.13 (0.94-1.35) | 2.26 (1.29-3.99) | 1.24 (0.46-3.37) |
| All models adjusted for age at diagnosis, ADG comorbidity, income quintile, rurality, year of diagnosis  *Model also adjusted for gender  PUG – psychiatric utilization gradient | | | | |
