# Supplementary material for: The impact of psychiatric utilisation prior to cancer diagnosis on survival of solid organ malignancies
Source: Br J Cancer. 2019 Mar 6;120(8):840–7. doi: 10.1038/s41416-019-0390-0 (PMC6474265; doi:10.1038/s41416-019-0390-0)
Supplement: Supplementary file 4 — Supplementary Table 4 [file 41416_2019_390_MOESM4_ESM.docx]

**Supplementary Table 4:** Anatomic site-specific multivariable Cox proportional hazards analysis for ACM assessing the effect of psychiatric utilization

|  | **HR (95%CI)** | | | |
| --- | --- | --- | --- | --- |
| **Anatomic Site** | **PUG Score 0** | **PUG Score 1** | **PUG Score 2** | **PUG Score 3** |
| Prostate | Ref | 1.03 (1.01-1.05) | 1.64 (1.47-1.83) | 2.58 (2.23-2.99) |
| Breast | Ref | 1.03 (1.01-1.05) | 1.34 (1.22-1.46) | 1.98 (1.78-2.20) |
| Lung* | Ref | 1.00 (0.99-1.02) | 1.19 (1.13-1.25) | 1.30 (1.22-1.39) |
| Colorectal* | Ref | 1.05 (1.04-1.07) | 1.31 (1.22-1.42) | 1.91 (1.73-2.10) |
| Melanoma* | Ref | 1.05 (1.01-1.08) | 1.54 (1.32-1.79) | 2.09 (1.72-2.53) |
| Thyroid* | Ref | 0.94 (0.86-1.02) | 1.80 (1.29-2.51) | 3.89 (2.74-5.53) |
| Bladder* | Ref | 1.06 (1.03-1.09) | 1.35 (1.17-1.56) | 1.73 (1.36-2.21) |
| Endometrial | Ref | 0.97 (0.93-1.01) | 1.23 (1.01-1.49) | 2.41 (1.84-3.15) |
| Kidney* | Ref | 0.97 (0.93-1.01) | 1.24 (1.06-1.44) | 1.49 (1.18-1.89) |
| Oral* | Ref | 1.06 (1.01-1.12) | 1.80 (1.49-2.18) | 2.07 (1.68-2.56) |
| All models adjusted for age at diagnosis, ADG comorbidity, income quintile, rurality, year of diagnosis  *Model also adjusted for gender  PUG – psychiatric utilization gradient | | | | |
