# Supplementary material for: The impact of psychiatric utilisation prior to cancer diagnosis on survival of solid organ malignancies
Source: Br J Cancer. 2019 Mar 6;120(8):840–7. doi: 10.1038/s41416-019-0390-0 (PMC6474265; doi:10.1038/s41416-019-0390-0)
Supplement: Supplementary file 5 — Supplement [file 41416_2019_390_MOESM5_ESM.docx]

**Supplement**

*A1. Coding scheme for identifying male cancer patients in the Ontario Cancer Registry*

Prostate cancer (ICD-9: 185; ICD-10: C61; ICD-O: C61.9)

Colorectal cancer (ICD-9: 153.8, 153.9, 154.0, 154.1; ICD-10: C18.9; ICD-O: C18.0-C18.9, C19.9, C20.9, C21.0-C21.2, C21.8)

Melanoma (ICD-9: 172.9; ICD-10: C43; ICD-O: C44.0-C44.9, C60.0-C60.2, C60.8, C60.9, C63.2)

Lung cancer (ICD-9: 162; ICD-10: C34; ICD-O: C33.9, C34.0-C34.3, C34.8, C34.9)

Bladder cancer (ICD-9: 188; ICD-10: C67; ICD-O: C67)

Thyroid cancer (ICD-9: 193; ICD-10: C73; ICD-O: C73.9)

Kidney cancer (ICD-9: 189.0, ICD-10: C64.9; ICD-O: C64.9)

Oral cancer (ICD-9: 145.9; ICD-10: C06.9; ICD-O: C00.3-C00.6, C00.8, C00.9, C01.9, C02.0-C02.4, C02.8, C02.9, C03.0, C03.1, C03.9, C04.0, C04.1, C04.8, C04.9, C05.0-C05.2, C05.8, C05.9, C06.0-C06.2, C06.8, C06.9)

*A2. Coding scheme for identifying female cancer patients in the Ontario Cancer Registry*

Breast cancer (ICD-9: 174.9; ICD-10: C50; ICD-O: C50)

Colorectal cancer (ICD-9: 153.8, 153.9, 154.0, 154.1; ICD-10: C18.9; ICD-O: C18.0-C18.9, C19.9, C20.9, C21.0-C21.2, C21.8)

Melanoma (ICD-9: 172.9; ICD-10: C43; ICD-O: C44.0-C44.9, C51.0-C51.2, C51.8, C51.9)

Lung cancer (ICD-9: 162; ICD-10: C34; ICD-O: C33.9, C34.0-C34.3, C34.8, C34.9), bladder cancer (ICD-9: 188; ICD-10: C67; ICD-O: C67)

Endometrial cancer, (ICD-9: 182.0; ICD-10: C54.1-C54.3, C54.9; ICD-O: C54.0-C54.3, C54.8, C54.9, C55.9)

Thyroid cancer (ICD-9: 193; ICD-10: C73; ICD-O: C73.9)

Kidney cancer (ICD-9: 189.0, ICD-10: C64.9; ICD-O: C64.9)

Oral cancer (ICD-9: 145.9; ICD-10: C06.9; ICD-O: C00.3-C00.6, C00.8, C00.9, C01.9, C02.0-C02.4, C02.8, C02.9, C03.0, C03.1, C03.9, C04.0, C04.1, C04.8, C04.9, C05.0-C05.2, C05.8, C05.9, C06.0-C06.2, C06.8, C06.9)

*B1. Validated ICES databases used*

The Canadian Institute for Health Information Discharge Abstract Database, which contains records for all hospitalizations;^1^ the Canadian Institute for Health Information National Ambulatory Care Reporting System, which contains records for emergency department visits; the Ontario Mental Health Reporting System, which contains records for psychiatric hospital admissions; the Ontario Health Insurance Plan database, which tracks claims paid for physician billings, and out-of-province providers (physicians, allied health, and hospitals);^2^ the Ontario Cancer Registry (OCR), a population-based registry estimated to be greater than 95% accurate and complete;^3^ and the Registered Persons database for demographic information.^4^

References

1. Juurlink, D. N., Preyra, C. & Croxford, R. Canadian Institute for Health Information Discharge Abstract Database: A Validation Study (Institute for Clinical Evaluation Sciences, Toronto, 2006).

2. Williams, J. I. & Young, W. A summary of studies on the quality of health care administrative databases in Canada. Can. Med. Assoc. 1, 339–345 (1996).

3. Robles, S. C., Marrett, L. D., Clarke, E. A. & Risch, H. A. An application of capturerecapture methods to the estimation of completeness of cancer registration. J.Clin. Epidemiol. 41, 495–501 (1988).

4. Iron, K., Zagorski, B. M., Sykora, K. & Manuel, D. G. Living and Dying in Ontario: An Opportunity for Improved Health Information. ICES Investigative Report (Institute for Clinical Evaluative Sciences, Toronto, 2008).
